# Very-Low-Dose Radiation and Clinical Molecular Nuclear Medicine

**DOI:** 10.3390/life12060912

**Published:** 2022-06-17

**Authors:** Chi-Jung Tsai, Kang-Wei Chang, Bang-Hung Yang, Ping-Hsiu Wu, Ko-Han Lin, Ching Yee Oliver Wong, Hsin-Lun Lee, Wen-Sheng Huang

**Affiliations:** 1 Department of Nuclear Medicine, Taipei Medical University Hospital, Taipei 110, Taiwan; 211010@h.tmu.edu.tw; 2 Taipei Neuroscience Institute & Laboratory Animal Center, Taipei Medical University, Taipei 110, Taiwan; kwchang@tmu.edu.tw; 3 Department of Nuclear Medicine, Taipei Veterans General Hospital, Taipei 112, Taiwan; bhyang@vghtpe.gov.tw (B.-H.Y.); khlin3@vghtpe.gov.tw (K.-H.L.); 4 Department of Radiation Oncology, Taipei Medical University Hospital, Taipei 110, Taiwan; wupinghsiu@tmu.edu.tw; 5 Department of Radiology, School of Medicine, College of Medicine, Taipei Medical University, No. 250, Wu-Hsing Street, Taipei 110, Taiwan; 6 Department of Radiology, University of Southern California, Los Angeles, CA 90007, USA; wongcy2@Sutterhealth.org; 7 Department of Nuclear Medicine, Cheng-Hsin General Hospital, No. 45, Cheng-Hsin Street, Beitou District, Taipei 112, Taiwan

**Keywords:** nuclear medicine, radionuclides, therapy, radiation, risks, protection

## Abstract

Emerging molecular and precision medicine makes nuclear medicine a *de facto* choice of imaging, especially in the era of target-oriented medical care. Nuclear medicine is minimally invasive, four-dimensional (space and time or dynamic space), and functional imaging using radioactive biochemical tracers in evaluating human diseases on an anatomically configured image. Many radiopharmaceuticals are also used in therapies. However, there have been concerns over the emission of radiation from the radionuclides, resulting in wrongly neglecting the potential benefits against little or any risks at all of imaging to the patients. The sound concepts of radiation and radiation protection are critical for promoting the optimal use of radiopharmaceuticals to patients, and alleviating concerns from caregivers, nuclear medicine staff, medical colleagues, and the public alike.

## 1. Introduction

With the development of molecular and precision medicine, nuclear medicine represents a typical modality of molecular imaging in the era of target-oriented medical care. Nuclear medicine also fulfills the requirements of the 3R principle (reduction, refinement, and replacement) for translational medicine. Currently, its minimally invasive, dynamic, functional, and biochemical characteristics in evaluating human diseases make it a mainstream in medical management. Various radiopharmaceuticals have been used in practice for disease diagnosis and therapies; however, there have been concerns over the emission of radiation from the ligand-labeled radionuclides [1], resulting in potential adverse consequences believed by the public to supposedly be beneficial, i.e., wrongly assessing benefits vs. risks of imaging to the patients [2]. In response to these concerns, concepts of radiation and radiation protection are critical for optimal use of radiopharmaceuticals to patients, and educating the caregivers, nuclear medicine staff, medical colleagues, and the public.

## 2. The Perception of Irradiation

Irradiation exists naturally on earth consisting of cosmic origin, soil, food, or even within the human body. People have thought that natural radiation could be beneficial to humans, such as (Radisson) the radium hot spring in North America, which the Indians called “holy water” traditionally, the earliest “Longnice hot spring” (1907), and the globally recognized “Green Song Quan” in Beitou, Taiwan. The Beitou stone (Hokutolite) is the only mineral named according to the toponym in Taiwan, containing traces of radioactive radium. Such mineral was believed to promote human health, such as pain relief, dermatitis alleviation, etc. Interestingly, artificial Beitou stones have been manufactured due to the rarity of the authentic material, which are added into undergarments for sale. Further studies are warranted to observe the effects of natural vs. artificial irradiation as it appeared that radiation from nature was accepted positively by the public while the artificial radiation, including medical use, was negatively perceived. Notably, the average amount of natural radiation in Taiwan is approximately 1.62 mSv per person per year [3,4]. People are exposed to an average annual background radiation level of about 3 mSv in the United States [5], about 2 mSv in Japan, and an average of 2.4 mSv throughout the rest of the world, with the highest dose of 7.5 mSv in Finland, based on data from the World Nuclear Association [6]. The effective dose of human exposure from a chest X-ray is about 0.1 mSv (the same as the typical dose received during a flight from New York to Tokyo) and a whole-body CT scan is about 10 mSv (typical chest CT, 7 mSv) [6]. Medical exposure in Japan is approximately 4 mSv per year.

On the other hand, as reported by the National Atomic Energy Council, R.O.C., except for more than 7000 chemical substances including hundreds of biologically hazardous materials and over 69 carcinogenetic substances, smoking 30 cigarettes per day is equivalent to 13 mSv of radiation per year, or 1 mSv per 2.3 cigarettes per day [7]. Polonium (Po)-210 in phosphate fertilizer for tobacco leaf growth is the radiation source of cigarette smoking. However, this is considered natural radiation and beneficial for health [8].

Theoretically, physical characteristics such as atomic weight, decay half-life, and energy of radioactive elements in the periodic table are inherent and hardly affected by external environments, such as temperature, humidity, pressure, or pH value, etc. Effects of radioactive elements on humans, either natural or artificial, may have identical physical characteristics.

**Figure 1:**
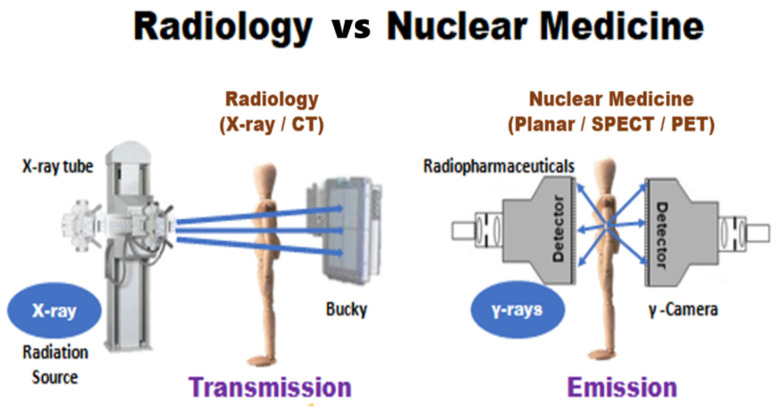
Different radiation mechanisms of imaging formation between radiology and nuclear medicine departments are shown, for which ways of radiation protection could be different accordingly.

Two major forms of radiation energy are employed in medical imaging: one is transmission radiation used in both radiology and radiation oncology treatment planning using the external beam, and the other is emission radiation used in nuclear medicine and brachytherapy planning (Figure 1). Therefore, radiation protection should be different between transmission and emission radiation. Sealing doors and evacuating staff in work places are mandatory for transmission radiation while keeping a distance from the source and minimizing the administration dose to meet the “As-Low-As-Reasonably-Achievable” (ALARA) principle, although the administrated doses are usually prescribed as the minimally required dose for imaging. Health physicians and nuclear medicine experts should execute such concept and raise awareness among other medical staff and the public. If the radiation issue in nuclear theragnostics is well-communicated and understood, clinical applications of nuclear medicine can be appropriately used, and patients can receive the optimal benefit from the contemporary nuclear theragnostics.

## 3. Consensus of the Low-Dose Radiation (LDR)

Data from the dose–response relationship for cancer mortality among A-bomb survivors suggested that the low-dose irradiation was set as 200 mGy by the 1993 report of the United Nations Scientific Committee on the Effects of Atomic Radiation (UNSCEAR) [4,9]. However, subsequent scientific surveys showed no statistical risk significance to the health impact compared to the healthy controls at doses of ≤100 mSv [10]. Thus, the definition of low-dose radiation as ≤100 mSv was proposed by The Biological Effects of Ionizing Radiation (BEIR) VII report of the US National Academy of Sciences [11]. The contemporary molecular nuclear medicine imaging could be categorized as very-low-dose radiation (VLDR) based on the dose calculation using the nuclear medicine radiation dose tool provided by The Society of Nuclear Medicine [12].

## 4. Healthy Effects of LDR

In contrast to high-dose radiation causing “non-stochastic or deterministic effects” such as acute radiation syndrome, skin rash, or cataract, healthy effects of LDR are mainly related to the “stochastic effect” to humans, such as cancers (involving somatic cells) or genetic (involving germ cells) disorders, that was supposed to have no threshold. A comprehensive biological and epidemiological study and survey [9] showing a statistically significant increase for cancers has hardly been described with LDR. The linear no-threshold (LNT) model is simple and makes it easy to understand the dose–response relationship for radiation physicians and the general population, yet epidemiological studies appeared insufficient to elucidate the LNT model in LDR. Therefore, the health effects of LDR and its interplay with confounding factors and the mechanisms of radiation carcinogenesis still warrant further evaluation [13].

Interestingly, a recent paper regarding trends of thyroid cancer published in JAMA 2017 showed that not only did micro-cancer incidence increase over the years but also the incidence of larger than 4 cm thyroid cancer and distant metastases [14]. The authors implied that overdiagnosis per se could not be fully explained, yet other factors such as obesity, smoking, and average radiation exposure, etc., needed to be considered. Radiation issues have always been a concern and thought to be relevant to the occurrence of thyroid cancer. It was reported that the average radiation exposure has increased over the years worldwide, mainly due to medical use [13], and accordingly, it was suggested to be responsible for the increased incidence of thyroid cancer. However, further molecular tests showed that the radiation-related somatic mutations, e.g., RET/PTC rearrangement, were decreased, while the non-radiation-related point mutations, e.g., BRAF or RAS, were increased [15,16].

Another article reviewing the rationale of LNT theory has challenged the long-term follow-up of cancer incidence of irradiated residents of Taiwanese radio-contaminated buildings, contradicting the LNT model or even in line with radiation hormesis [17,18].

## 5. Very-Low-Dose Radiation (VLDR) of Nuclear Medicine Imaging

The 2017 ASNM Management Meeting Minute in Shanghai declared that contemporary nuclear medicine is to use low dosages of medical radiation for patient care. Facts of low-dose radiation including: (1) Credible evidence of imaging-related low-dose (100 mGy) carcinogenic risk is nonexistent. (2) It is a hypothetical risk derived from the evidently false linear no-threshold (LNT) hypothesis. On the contrary, low-dose radiation does not cause, but more likely helps prevent cancer. (3) LNT and its offspring, ALARA, are fatally flawed, focusing only on molecular damage while ignoring protective, organismal biologic responses. Accordingly, the meeting concluded as follows: (1) Medical imaging-related radiation will produce more benefit than harm to the exposed patients. (2) It may have no upper limit as it is far away from that. (3) Medical radiation is not natural radiation. (4) Patients are not representative of the general population. (5) The medical radiation is “low-dose” if the patient can obtain benefits from it. (6) The upper limit of a “low-dose” is not the same for all people, and it should be personalized. (7) Much research has proven that there needs to be more effort in gaining support from the government and educating the general public regarding the proper attitude toward LDR or VLDR. 

The above-mentioned declaration was thereafter emphasized in the AOCNMB 2019 Meeting in Shanghai as “The inheritance of Shanghai Manifesto 2016”. The important points included: (1) Nuclear medicine should not be limited to only medical imaging but also radionuclide therapy, which is aimed towards systemic α- and β-particle therapies and local boron neutron-captured therapy (BNCT), based on hybrid molecular imaging. (2) Nuclear medicine should not be limited to only SPECT and PET and should move towards hybrid molecular imaging involving SPECT-CT and PET-CT. (3) Nuclear medicine is a safe medical approach with low radiation exposure, and it is patient-, medical staff-, and environment-friendly [19]. Despite technological and pharmaceutical improvements and innovation, the radiation safety issue is the fate we must face, especially in the era of media and knowledge explosion.

Nuclear medicine imaging belongs to the low-dose/low-dose-rate radiation exposure. Patients who received trace amounts of radioactive material (i.e., the radioligand or radiopharmaceutical) need to be amplified more than 1000 times by the emitted photoelectrons through photomultiplier (PMT) tubes to acquire enough counts to be effectively analyzed and transferred into a clinically interpretable image. Our recent survey used 51 thermoluminescence dosimeters (TLD) (one for background control) for a radiation survey of areas in hospital wards that carried relatively high volumes of nuclear medicine examinations, such as cardiovascular or genitor-urological units, to assess possible radiation exposure to the workers in those wards. The environmental TLD data all showed a background value. A similar personal survey in the Far-East Medical Center also indicated that routine nuclear medicine examinations did not expose the caregivers to extra radiation. In another study performed in a university hospital in Saudi Arabia, the annual average effective doses for diagnostic radiology, nuclear medicine, and radiotherapy workers (total of 100 persons) were found much below the international recommended dose limit of 20 mSv (0.66, 1.56, and 0.28 mSv, respectively) [20].

The results might alleviate hospital colleagues’ concerns of caring for the patients who underwent nuclear medicine examinations.

From the scientific point of view, a systemic study using an extraordinarily sensitive assay system, such as a fluorescence microscopic detecting discrete foci of 53BP1 protein (which is one of the DNA damage response factors), detected that DNA double-strand breaks (DSBs) caused by low-dose radiation rarely occurred.

The effects of radiation cellular effects could be roughly divided into indirect and direct actions.

The indirect action was mainly related to those of radiation with low linear energy transfer (LET), such as X- or γ-ray, producing free radicals to cause cellular damage indirectly. Indirect DNA damage is caused by free radicals or reactive oxygen species (ROS) generated from the interaction of a secondary electron with a water molecule. Notably, such action could be affected by the microenvironmental oxygen content, which might play an important role as a radiosensitizer in humans owing to approximately 70% of the cell being composed of water [21]. Notably, the total number of free radicals yielded was determined by the total radiation dose [22]. Thus, in the case of DSB, the number of DSBs produced also depends on the total dose.

In contrast, those with a high LET effect such as α-, proton, fast-neutron, or heavy-ion particles carry out a direct radiation action, mainly resulting in DSB. Direct DNA damage occurs when incoming radiation excites or ionizes the target molecule to release the secondary electron, and the secondary electron then interacts with DNA directly, leading to a series of biological changes. DSB could lead to irreversible DNA damage, and it is therefore considered the main cause of cell death, carcinogenesis, and other mutations. Suzuki et al. demonstrated that 100 mGy administered at a low-dose-rate did not result in any excess DSBs. In their observations, four DSBs/cell were induced after an acute 100 mGy of radiation, whereas a yearly (chronic) 100 mGy dose induced about 0.01 DSB/cell/day, compared to 0.1 DSB/cell/day by the naturally endogenous ROS of the human body [9]. The endogenous DSBs from single-strand DNA lesions in replicating cells were equivalent to those of radiation at a dose rate of 282 mGy/h [9]. The observations emphasized the importance of the dose rate for cancer risk. This report also concluded that, with low-dose or chronic exposure to low-LET irradiation, the risk of adverse heritable health effects to children conceived after their parents were exposed is small compared to baseline frequencies of genetic diseases in the population.

**Figure 2:**
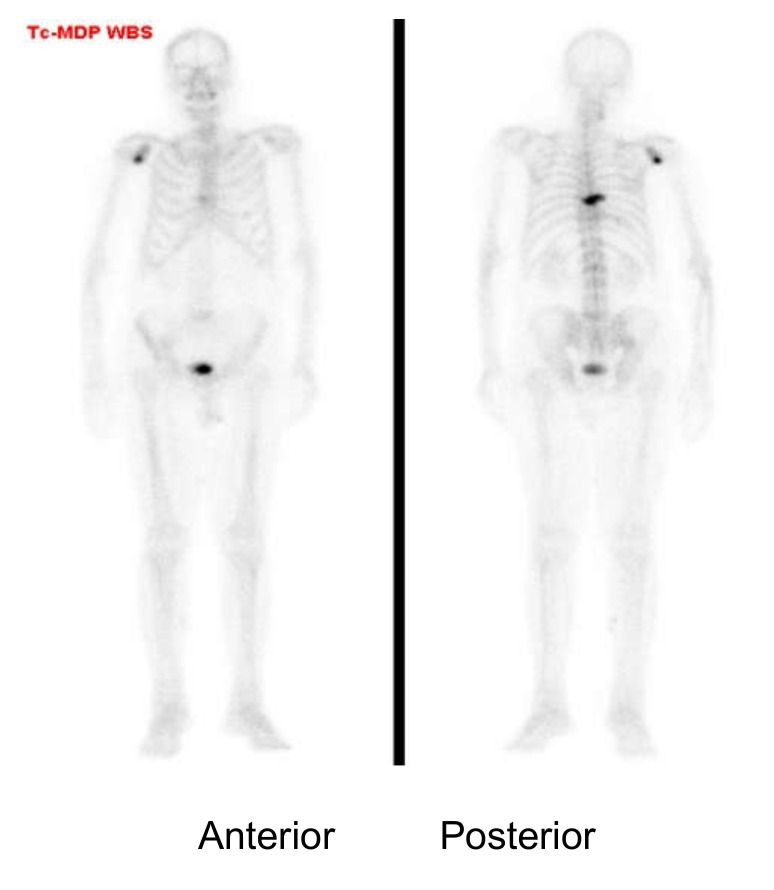
This was an 88-year-old man with prostate cancer and bony metastases presenting right shoulder and T-spine uptake. The T-spine uptake was clearly seen in the posterior view but not well seen in the anterior view. The uptake in the right shoulder and the bladder showed less differences between the two views owing to less tissue penetration and nearly the same distance between the two camera heads. This representative imaging illustrated the fact that radiation penetration in the human body used in routine nuclear medicine examinations appeared not as high as the public expectation [24].

Radiation in nuclear medicine examinations belongs to the type of “low dose with low dose rate”. In clinical practice, the dose rate is 6.9 μSv/h at 1 m apart and 2.6 μSv/h at 2 m apart immediately after injection of 20 mCi Tc-99m MDP for a bone scan. The dose reduced to 3.6 and 1.4 μSv/h, respectively, 2 h after injection (Figure 2). For those with 10 mCi of F-18 FDG PET, it is 29 μSv/h at 1 m apart and 11 μSv/h at 2 m apart immediately after injection, and the dose reduced to 8.6 and 3.4 μSv/h, respectively, 2 h after injection. The dose rates are much lower than the recommended restrictions (70 μSv/h at 1 m) [23]. A website provided by The Society of Nuclear Medicine, USA, is readily available to estimate radiation doses of patients who received routine nuclear medicine examinations by inputting the types of radionuclide and the administrated radioactivity (SNM homepage, category: Nuclear medicine radiation dose tool) [12].

## 6. Controversy in Medical LDR

**Figure 3:**
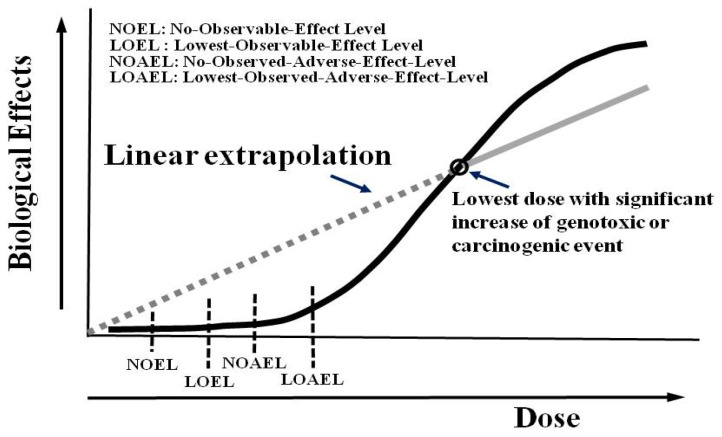
The representative dose–response relationship in assessing the biological effects of harmful substances. For those with potentially genotoxic and carcinogenic effects, a linear no-threshold (LNT) curve was proposed owing to the difficulty to assume a threshold of the effect (adapted from [29]).

It has long been recognized that radiation doses over 100 mSv will induce tissue adverse effects proportional to the exposed doses (namely, non-stochastic effect or deterministic effect), such as acute radiation syndrome, skin erythematous change, or cataract, etc. This portion of radiation doses revealed a linear relationship with detrimental health effects [25]. On the other hand, doses less than 100 mSv, so-called low-dose irradiation, are probably related to stochastic effects, such as cancers or genetic effects. Hypotheses regarding effects of ionizing radiation below this level in humans include the linear no-threshold (LNT), hormesis [26,27], and linear with threshold (LT). The latter is commonly applied in non-carcinogenic and genotoxic chemical or toxic events, with people exposed below this level showing no visible biological effects or equivalent to normal biological variances. As for the concept of radiation hormesis, proposing that a little radiation might be good for health, it is based on the adaptive response of cells and organisms to low levels of radiation by the stimulation of repair mechanisms. The atomic bomb survivors and former workers in nuclear industries also revealed the possibility of hormesis. Interestingly, a recent report declared that low-dose radiation can ease the disease course and reduce the need of intensive care for COVID-19 patients [28]. Despite this, most of the general public still believe the LNT model because it is easy to assume the potential effects of low-dose irradiation in human beings. From a radiation protection point of view, the LNT model forces the involved parties to make their best efforts to apply the ALARA principle more effectively and reminds us of the concept of “no absolutely safe radiation” [29]. Unfortunately, there is still no data robust enough to strike down the LNT hypothesis. Therefore, the use of LNT will likely continue. Of note, LNT is usually derived from an extrapolation of the lowest dose with a significant increase of an event such as genotoxic or carcinogenic incidence, as shown in Figure 3 [29].

The biological effect within the internally extrapolated range is still lacking solid scientific data, meaning that there is controversy in the effects of low-dose radiation in human beings. Although epidemiological studies have failed to prove that hormesis exists, “they failed to show radiation in American homes causes cancer” [26], epidemiology may be an inexact science [30], “It’s irrelevant whether it’s real or not. What is real is that there is no demonstrable injury due to radiation at low or even moderate levels” (Stanley J Goldsmith, M.D., Cornell Med. Center). The National Academics, an adviser on science, engineering, and medicine, reported the BEIR VII (health risks from exposure to low levels of ionizing radiation) in 2005 and suggested to retain the LNT hypothesis, based on safe considerations: “Until someone can clearly demonstrate that LNT is not true, we are going to have to make that assumption” (Charles Meinhold, the previous president of the NCRP). A recent petition to end the reliance on the LNT model rejected by the Nuclear Regulatory Commission (NRC) was also based on the same assumption [31].

Given the lack of scientific consensus about potential risks from low doses of radiation, a position statement on radiation risks from Medical Imaging Procedures from the American Association of Physicists in Medicine (AAPM) in April 2018 said that predictions of hypothetical harm from the use of diagnostic imaging are highly speculative. The AAPM and other radiation protection organizations specifically discourage these predictions, which can lead to “sensationalistic stories” in the public media, causing patients to fear or refuse safe and appropriate medical imaging, which is deemed to be to the detriment of the patient [32].

## 7. Nuclear Medicine Molecular Radionuclide Treatment

As well as diagnostic nuclear medicine, nuclear molecular radio-treatment rapidly emerged. “Theragnostics” has become a popular form of treatment, especially in the diagnosis and therapy of patients with neuroendocrine tumors and prostate cancers, owing to the promising patient outcomes fueled by PET imaging using NETSPOT™ (68Ga-DOTATATE) by Novartis from Advanced Accelerator Applications due to its clinical impact. Radio-iodine-131 treatment of differentiated thyroid carcinoma (DTC) is a commonly used procedure in practice and probably the first clinically applied “Theragnostics”. While empiric approaches for prescribed activities of 131-I have been widely used, dosimetry evaluation of the radiation absorbed dose to the cancer targets and critical organs, such as the blood and bone marrow with extraordinary doses of I-131 therapy, >200 mCi, poor renal or pulmonary functions, or potential bone marrow abnormalities, are also suggested [33,34].

Efficacy and safety are essential for therapeutic pharmaceutical marketing. The most common concern for therapeutic radiopharmaceuticals is the radiotoxicity to critical organs such as bone marrow and kidneys. Thus, internal dosimetry is needed. However, the dosimetry-guided approach is a time-consuming and complex procedure [34] and potential inaccuracies in calculating doses delivered to the tumor (such as partial volume effect, microenvironment, etc.) and normal organs often make clinical dosimetry suboptimal. With the emergence of novel and effective radionuclides in “theragnostics” and precision medicine, dosimetry calculation, however, may become crucial in contemporary medical care, in that “one dose may not fit all”. The advantages of the dosimetry-based approach are: (1) optimization of radionuclide therapy, (2) estimation of the cost-effectiveness ratio of the treatment in a single patient, (3) minimization of risks of toxicity, and (4) individualization according to clinical needs (eradication, palliation) [35]. For example, dosimetry-based PRRT using Lu-177 Octreotate with four standard cycles might deliver 23 Gy to kidneys and 2 Gy to bone marrow (although dosimetry is not the same for all patients, likely related to an individual genetic basis). Dosimetry may guide optimal treatments and enable us to realize who may require fewer cycles and who can tolerate more cycles [36]. Sandström et al. concluded that individualized absorbed doses were essential for optimization [37], and prospective dosimetry based on a 23 Gy threshold for Lu-177 [38] and 37 Gy for Y-90 DOTATOC [39] is feasible to reduce renal toxicity. ^177^Lu-PSMA (a beta emitter on prostate-specific membrane antigen) therapy efficacy was assessed by using prospective trials, meta-analyses, and major retrospective trials [40] to be generally safe, with a low toxicity profile, which is a promising treatment in patients with metastatic castration-resistant prostate cancer with good clinical efficacy [40]. Similarly, Radium Ra-223 dichloride (radium-223, Xofigo^®^) is a targeted alpha therapy also approved for the treatment of castration-resistant prostate cancer with symptomatic bone metastases and no known visceral metastatic disease [41]. Radium-223 provides a new treatment option, with evidence of a significant survival benefit, both in overall survival and in the time to the first symptomatic skeletal-related event [41].

While simplified methods for clinical dosimetry are still moving forward [42,43] to identify lesions or patients that would benefit from treatment, they exclude treatment of lesions without benefit. They also include those additional treatments that should be needed. Thus, a tumor irradiation dose estimation might be desirable.

## 8. Lessons from Chernobyl and Fukushima Accidents

When nuclear power plants catastrophically fail, it can cause vast human and environmental damage. Radiation releases from nuclear accidents are far beyond any controlled medical radiation and technically cannot be contained in a space and will not stop at national borders, potentially creating disastrous radiation damage. However, all important radionuclides, such as I-131, Cs-137, Sr-90, and Pu-239/-240, were monitored and data were collected in both the 2011 accident at the Fukushima Daiichi nuclear energy facility in Japan and the Chernobyl accident in the former Soviet Union in 1986. According to the International Atomic Energy Agency (IAEA), there was less total atmospheric release of radioactivity from the Fukushima accident compared with Chernobyl due to the different accident scenarios and mechanisms of radioactive release. With model simulations and assumptions, the total activity released in Chernobyl was estimated as 5.3 × 10^18^ Bq [44]. The total activity released in the Fukushima accident was 10–15% of the Chernobyl value (5.2 × 10^17^ Bq) [44]. No deaths from radiation exposure have been attributed to the accident at Fukushima, as published in 2013 by the World Health Organization [45]. It also concluded that health risks from radiation released during the Fukushima accident were minimal, with essentially no health effects outside Japan, suggesting improvement in countermeasures.

## 9. Conclusions

Although the commonly defined low-dose radiation is no more than 100 mSv, radiation doses of nuclear imaging used in clinical practice are far below the level and are easily detected and monitored. All clinical applications are approved and regularly inspected by governmental authorities such as the Taiwan Food and Drug Administration (TFDA) and the Atomic Energy Council (AEC). On the other hand, theragnostics is a newly emerged medical technology enabling advanced cancer patients to be treated precisely and effectively. The safety of such a high therapeutic dose has become an important issue in clinical care. Internal dosimetry on an individualized basis seems to be clinically needed. More scientific data regarding radiation in medical use and more communication to the medical staff and the public are warranted to optimize the benefit of medical radiation in clinical services.
